# Distinct disease phenotypes linked to different combinations of GAA mutations in a large late-onset GSDII sibship

**DOI:** 10.1186/1750-1172-8-159

**Published:** 2013-10-10

**Authors:** Simone Sampaolo, Teresa Esposito, Olimpia Farina, Daniela Formicola, Daria Diodato, Fernando Gianfrancesco, Federica Cipullo, Gaetana Cremone, Mario Cirillo, Luca Del Viscovo, Antonio Toscano, Corrado Angelini, Giuseppe Di Iorio

**Affiliations:** 1 Department of Medical Sciences, Surgery, Neurological, Metabolic and Aging, Second University of Naples, Naples, Italy; 2 Institute of Genetics and Biophysics “Adriano Buzzati-Traverso”, National Research Council of Italy, Naples, Italy; 3 Department of Clinical and Experimental Internal Medicine, Second University of Naples, Naples, Italy; 4 Department of Neuroscience, University of Messina Italy, Messina, Italy; 5 IRCCS San Camillo, Lido Venice, Italy

**Keywords:** Pompe disease, GAA gene, Mutation analysis, Genotype-phenotype correlations

## Abstract

**Background:**

Glycogenosis type II (GSDII or Pompe disease) is an autosomal recessive disease, often characterized by a progressive accumulation of glycogen within lysosomes caused by a deficiency of α-1,4-glucosidase (GAA; acid maltase), a key enzyme of the glycogen degradation pathway. To date, more than 326 different mutations in the *GAA* gene have been identified in patients with GSDII but the course of the disease is difficult to be predicted on the basis of molecular genetic changes. Studies on large informative families are advisable to better define how genetics and non genetics factors like exercise and diet may influence the clinical phenotype.

**Methods and results:**

In this study, we report on clinical, instrumental, and pathological features as well as on molecular analysis of a family with 10 out of 13 siblings affected by late-onset Pompe disease. Three mutations segregated in the family, two of which are novel mutations. Siblings showing a more severe phenotype were compound heterozygous for c.118C > T [p.R40X] and c.2647-7G > A [p.N882fs] on *GAA*, whereas, two patients showing a mild phenotype were compound heterozygous c.2647-7G > A [p.N882fs] and c.2276G > C [p.G759A] mutations. Quantitative expression analysis showed, in the patients carrying p.R40X/ p.N882fs, a significant (p 0.01) correlation between the levels of expression of the mutated allele and the age at onset of the disease.

**Conclusions:**

As far as we know, this is the largest informative family with late-onset Pompe disease described in the literature showing a peculiar complex set of mutations of *GAA* gene that may partially elucidate the clinical heterogeneity of this family.

## Introduction

Glycogenosis type II (GSDII, or Pompe disease; MIM 232300) is an autosomal recessive storage disorder due to mutations of the acid α-glucosidase gene (*GAA*) on the chromosome 17q21-23 which causes absent or deficient activity of the lysosomal enzyme acid α-glucosidase (GAA) [1]. GAA catalyzes the hydrolysis of α-1,4 and α-1,6 links of glycogen into the lysosomes; its activity reduction, below the critical threshold of approximately 35% of control levels, leads to a progressive accumulation of glycogen within these organelles, and, later, between myofibrils. GAA is ubiquitous in the body, but its shortening is particularly harmful in muscle tissues, and contributes to determine the clinical phenotype by cardiac, skeletal, and respiratory muscles involvement [1,2].

GSDII is clinically classified into classic infantile and late-onset forms. The classic infantile form is characterized by marked generalized muscle weakness and severe cardiomyopathy with rapid progression and a fatal outcome within the second year of life. Late-onset form comprises a wide spectrum of highly variable phenotypes which are dominated by limb-girdle, paraspinal, and respiratory muscles weakness [2].

To date, GSDII has been linked to more than 326 different mutations of the *GAA* gene including missense, nonsense, large and small insertions and deletions, as well as frame-shift and splicing changes, as listed in the latest update (25 June 2012) of the Erasmus Mutation Database. All these mutations occur in homozygous or compound heterozygous affected individuals and are associated with absence of GAA activity in the infantile form, and with a residual GAA activity ranging 3-35% of control values in the late onset forms. These data indicate a clear cut relationship between enzyme activity levels and severity of phenotype in the infantile form whereas, in late-onset patients, there is no definite correlation [3-9]. These evidences lead to hypothesize that the expression of *GAA* mutations could be modified by genetic background and non genetic factors (life-style, nutrition, and environment) [10,11]. Studies on informative families, where *GAA*-mutated members have the closest possible genetic background are advisable to better define the genotype-phenotype correlation. This could be particularly useful in families with a late-onset form in order to detect other relatives initially affected that could be early treated by Enzyme Replacemement Therapy (ERT), approved since 2006 either for children or for late onset form with effective results [12]. We report herein the detailed clinical, pathological, and molecular features of a large, informative Italian family with late-onset GSDII.

### Patients and methods

#### Case index

A 41-years-old man (II-7) was admitted to our Department complaining of progressive difficulties in walking, back pain, and easy fatigability since 3 years. His older brother II-2 was confined to the wheelchair since 6 years because of an undefined myopathy, while 11 other siblings were reported to be healthy. His father had died because of respiratory failure and his mother for myocardial infarction, at 69 and 71 years of age, respectively. Patient neurological examination showed waddling gait and hyperlordosis resulting from paravertebral and pelvic-girdle muscles weakness. Serum CK, LDH, ALT, and AST values were, respectively, 466, 180, 65, and 58 U/L, respectively. EMG revealed a myopathic pattern. Echocardiography disclosed aortic arch dilation.

**Figure 1:**
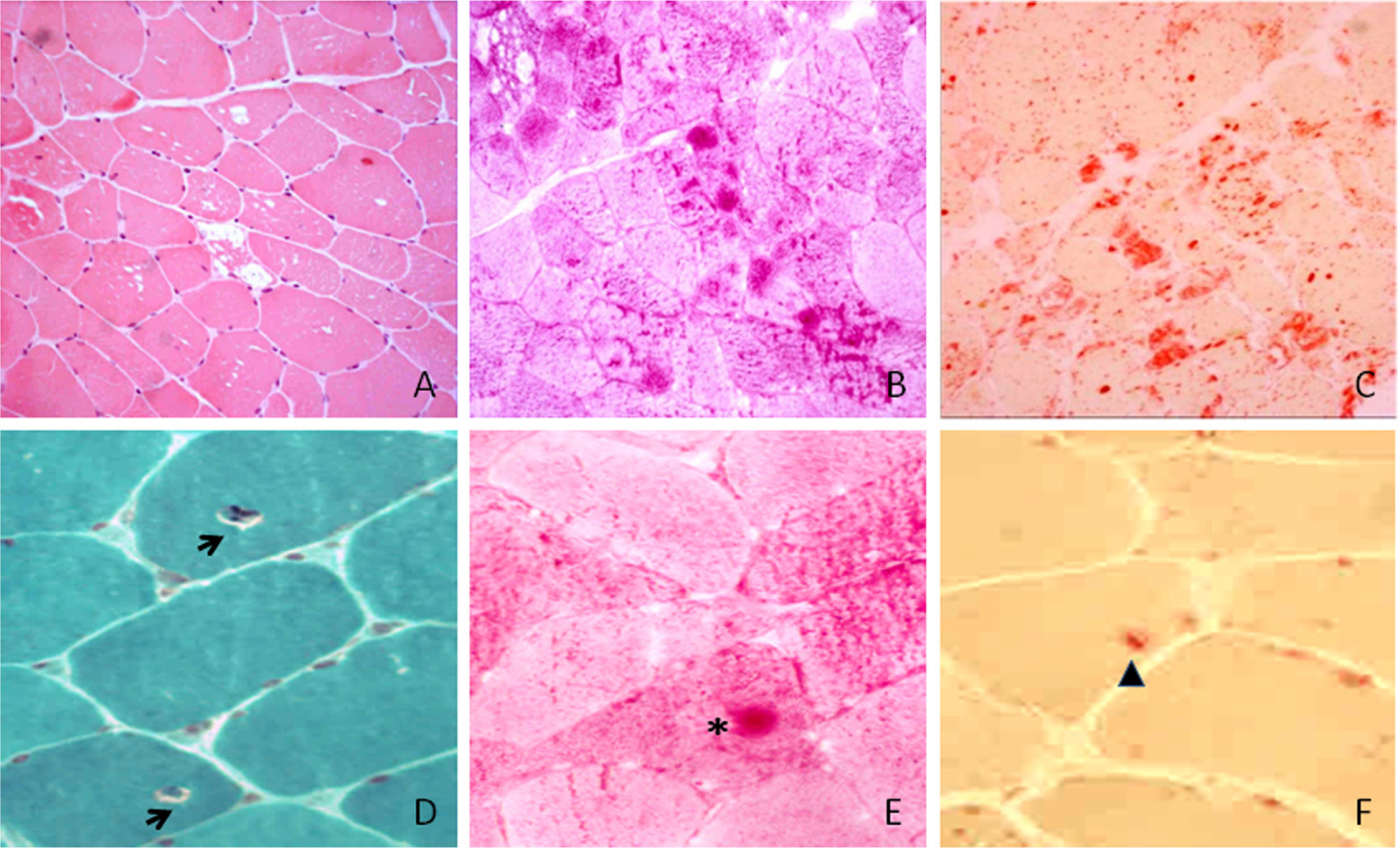
**Cryostatic sections of vastus lateralis muscle biopsies. A)** HE, with muscle fibres showing vacuoles of variable size, the vast majority of which contain PAS **(B)** and acid phosphatase **(C)** positive material. The muscle biopsy of the sibling II-8 disclosed peculiar microscopic changes consisting in thin gaps surrounding paracentral nuclei **(D**, Gomori, arrows**)** at level of which PAS **(E**, asterisk**)** and to a lesser extent acid phosphate **(F**, arrowhead**)** positive deposits were seen in some fibres. (Upper series 10×; lower series 20×).

Right vastus lateralis muscle biopsy was performed and muscle specimens processed with standard protocols for histological, hystochemical, and ultrustructural studies. These showing scattered intracytoplasmic vacuoles with basophilic amorphous materials inside, in addition to mild fiber size variation as well ultrastructural studies detecting a widespread fiber vacuolation with glycogen accumulation within lysosomes, suggested a diagnosis of GSDII disease (Figure 1) which was confirmed by GAA activity assay on muscle homogenate [13,14].

#### Family studies

**Figure 2:**
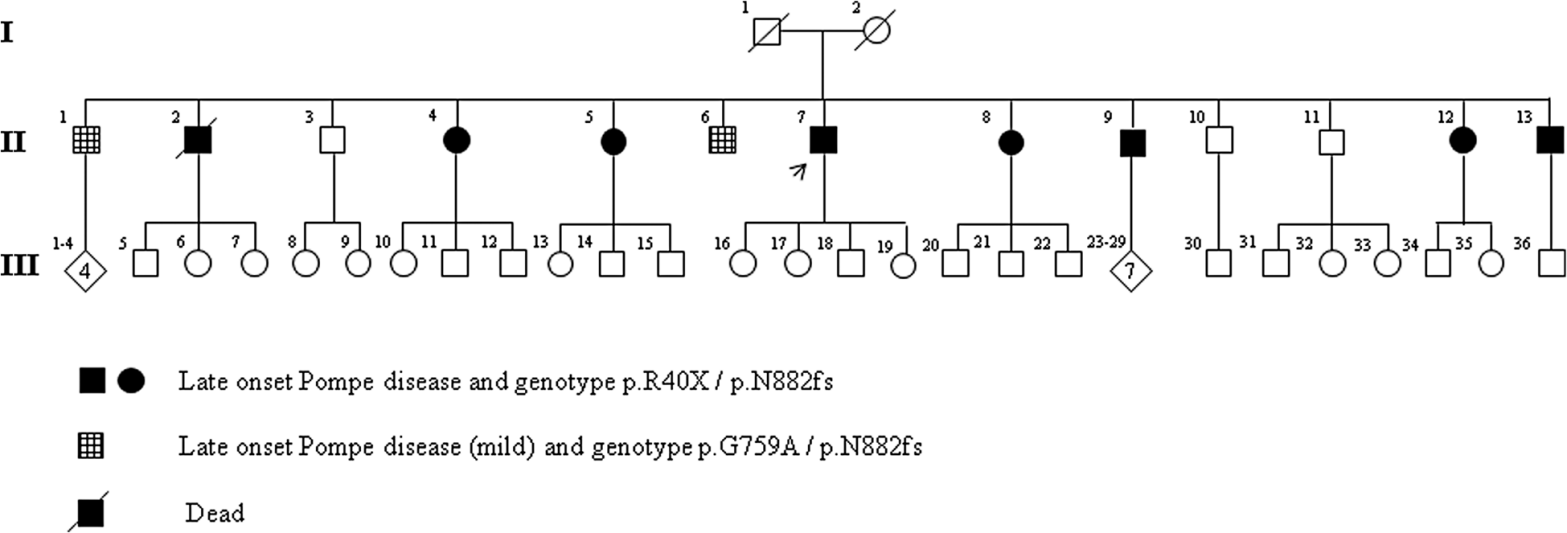
The complete pedigree of the Pompe Italian family.

The pedigree of this family (Figure 2) includes 13 siblings (9 males and 4 females) in the second generation born from non-consanguineous parents. Clinical data on the grandparents’ families and relatives were not available. The numerous descendants (36 individuals; age ranging 6–32 years) are reported to be healthy but we have not yet examined them all. Few weeks after the evaluation of the index case, his brother confined to a wheelchair (II-2) died suddenly of respiratory failure at 54 years of age. After this sad event, all of them decided to be clinically visited in our Department. To determine as precisely as possible their age at first symptoms onset, the period during which they started to experience impairment of motor functions in daily activity was asked. Informed consent was obtained from each patient. The study protocol conforms to the ethical guidelines of the 1975 Declaration of Helsinki, as reflected in the a priori approval by the institution's human research committee.

#### Clinical examination

10 out 11 siblings were screened collecting their clinical history and neurological examination. Skeletal muscle strength was evaluated by manual muscle testing (MMT) using the Medical Research Council (MRC) grading scale (range 0–5). Tongue strength was also assessed using the manual method described by Dubrovsky et al. [15].

Muscle functions were assessed using the Gait, Stairs, Gowers, Chair (GSGC) scale [16]. Functional endurance during prolonged ambulation was evaluated with the six-min walk test (6MWT).

*Respiratory function* was evaluated measuring forced vital capacity (FVC) with spirometry in the upright and supine positions [17,18]. Results were expressed as a percentage of predicted normal values. Values lower than 80% of predicted indicating variable degrees of restrictive respiratory disease, ranged from mild to very severe, according to American Thoracic Society standards [19].

Cardiac function was evaluated by clinical examination, baseline EKG and echocardiography.

Routine blood analyses were performed including CK, LDH, AST, and ALT, calcium, phosphorus, and magnesium levels as well as thyroid hormones concentrations and thyroid antibodies titers assessment.

#### Electrophysiological study

Concentric needle EMG was performed. Spontaneous activity was assessed at rest during insertion and tapping of the muscles. Motor unit potentials and activity pattern at maximal voluntary contraction were evaluated. The left deltoid, the right tibialis anterior and the abductor digiti minimi as well as paraspinal lumbar and dorsal muscles were studied.

#### Muscle histology and biochemistry

Open biopsy of the vastus lateralis of the right quadriceps muscle was performed, after informed consent, in 7 affected individuals (II-4, 5, 7, 8, 9, 12, 13) apart from II-1 and II-6 who refused the biopsy. Three muscle specimens from each biopsy were processed according to standard procedures for histology and biochemistry studies and for the extraction of nucleic acids.

#### Measurement of GAA activity

The GAA activity was determined on dried blood spots, in all siblings participating to the study, using both tandem mass spectrometry and fluorimetry [20,21]. Muscle residual GAA activity was also measured on homogenate of frozen muscle specimens in 7 affected siblings (II-4, 5, 7, 8, 9, 12, 13), according to the methods above mentioned [13,14].

#### Muscle imaging

Whole Body Muscle Magnetic Resonance Imaging (WB-M-MRI) was performed on a 1.5-Tesla (40 mT/min, max. - slew rate 200 T/m/s) whole-body scanner (Magnetom Avanto, Siemens Medical System, Erlangen, Germany) using GRAPPA algorithm as integrated Parallel Acquisition Technique (iPAT) to minimize acquisition time [22-24]. The scanner allows the connection of up to 76 receiver elements from multiple phased-array surface coils, covering the patient from head to toe, with simultaneous signal reception from 32 independent receiver channels. In the scanner, the patient was placed in the supine position with arms along the body and examined from head to ankles with T1 and T2 sequences at five body levels in the coronary orientation: head/neck, pelvis, thighs and ankles as well as the thorax/abdomen; Imaging parameters were TR/TE 577/11, 5 mm slices, 384 × 384 for T1 acquisition and TR/TE 5720/87, 5,00 mm slices, matrix 384 × 384 for T2 acquisition; prospective two-dimensional (2D) navigator correction of the respiratory phase (PACE, prospective acquisition correction) to avoid breath artifacts. Using PAT acceleration, whole-body STIR imaging was possible within 12:28 min and T1-weighted imaging within 16:10 min at a 1.3 × 1.1 mm and 1.8 × 1.3 mm in-plane resolution, respectively. For the coronal whole-body imaging a PAT-factor of 3 was used, except from the ankles that were acquired with a PAT factor of 2. After the examination, the images were electronically aligned to one image of the whole body in the coronal plane. Including localizer time, total acquisition time for WB-MRI was 45 min, mean room time was 54 min. The total imaging time was about 30 minutes. Before the examination, patient was requested to restrain from excessive ambulation or exercise because T2 sequences potentially being affected by exercise or activity [25].

#### Intracranial arteries study

Magnetic Resonance Angiography (MRA) was performed by Angio-RM TOF 3D axial Siemens Synphony 1.5 T to evaluated intracranial arteries for vessel wall ectasia and/or aneurysm. Vessel total square diameter was measured at the apex and middle third of the basilar artery and at the middle third of the intracranial portion of right and left internal carotid artery.

#### Bone mineral density (BMD)

Total body, lumbar, and femur scans were obtained by Dual x-ray absorptiometry (DXA model DPX-L, LUNAR Radiation, Madison, WI, USA). Acquired data were analyzed using the LUNAR Radiation body composition program to determine BMD for total body, arms, legs, and trunk. According to the World Health Organization standards results were scored as follows: BMD z score greater than − 1, between − 1 and − 2.5 and less than − 2.5 corresponding to normal, osteopenia, and osteoporosis, respectively [26].

#### Mutation analysis

Genomic DNA was isolated from blood lymphocytes of the patients, according to standard procedures. DNA was quantified and diluted at 20 ng/μL for the amplification by PCR.

The *GAA* gene (NG_009822.1) was amplified by PCR from genomic DNA. All exons and flanking intron sequences were amplified using specific primer pairs available on request. In a final volume of 25 μl, 100 ng of genomic DNA was combined with 0.5 μM of each primer, 0.2 mM all dNTPs, 1× buffer (20 mM Tris, 10 mM Hepes, 2.5 mM magnesium sulfate, 10 mM potassium chloride and 10 mM ammonium sulfate) (Fermentas) and 1U of Dream Taq (Fermentas). After polymerase activation for 3 min at 94°C, reactions were carried out for 30 sec. at 94°C, 30 sec. at melting temperature and 45 sec. at 72°C for 40 cycles and 8 min at 72°C for finally extension. PCR products were confirmed by electrophoresis in 1.5% agarose gel and sequenced [27].

#### Sequencing

PCR products where directly sequenced on both strands using the Big Dye Terminator Ready Reaction Kit (Applied Biosystems). Sequencing reactions were performed on a 9700 Thermal Cycler (Applied Biosystems) for 25 cycles of 95°C for 10 sec., TM for 5 sec. and 60°C for 2 min. After the sequencing, each reaction was column-purified (Amersham) to remove excess dye terminators, and was subsequently run on the ABI prism 3700 Genetic Analyser (Applied Biosystems). Sequences were analyzed using multiple alignments of sequences with the program Autoassembler (Applied Biosystems) [28].

#### mRNA extraction and cDNA sequencing

RNAs were isolated from muscle biopsies using TRIzol reagent (Invitrogen, Carlsbad, CA, USA) according to the manufacturer’s instructions, and dissolved in diethyl pyrocarbonate (DEPC)–treated water. 1 μg of total RNA extracted by skeletal muscle biopsies of seven siblings (II-4, 5, 7, 8, 9, 12, 13) and two control muscles from the Neuromuscular Tissues Bank of the Second University of Naples, was reverse transcribed with the Transcriptor HiFi cDNA Synthesis kit (Roche). For the amplification of the cDNA fragment comprising the alternative splice site, was used a forward primer designed in exon 18 combined with a reverse primer in exon 20 (forward: CACCATCAACGTCCACCTCC and reverse: GTGTCGGGGCTGTAGGTGAA). PCR amplification was performed in a final volume of 25 μl using 20 ng of single stranded cDNA, as described before.

#### Expression analysis of the mutated c.2647-7G > A transcript

To examine the expression pattern of the alternative transcript generated by the c.2647-7G > A mutation qPCR assay was performed using a specific oligonucleotide designed to detect only the alternative transcript. qPCRs were performed using 10 ng of cDNA, 0.5 μM of human GAA transcript-specific primers and 1× of FastStart Universal SYBR green Master Mix (Roche) in a total volume of 20 μl. Thermal cycling was performed using the LightCycler System DNA Engine Opticon 2 (MJ Research) with an initial denaturation of 10 min. at 95°C, followed by 40 cycles of 15 sec. at 95°C, 60 sec. at 60°C. Each reaction was performed in triplicate and PCR products were analyzed by electrophoresis on 3% agarose gels. The expressions were normalized versus ‘glyceraldehyde 3-phosphate dehydrogenase (GAPDH)’ to account for differences in starting material and cDNA reaction efficiency [28].

GAA transcript primers were forward primer 5′ TTCCTGGCCAGGAATCCCAG 3′and reverse primer 5′ GTGTCGGGGCTGTAGGTGAA 3′; the GAPDH primers were forward primer 5′AGCCACATCGCTCAGACAC 3′ and reverse primer 5′GATCTCGCTCCTGGAAGATG 3′.

## Results

### Clinical evaluation of the affected siblings

Main demographic and clinical data of all affected siblings are summarized in Table 1. The first symptoms for all affected siblings were fatigability and pain which were complained at mean age 43,2 ± 11,04 years (range 20–56 years), including in this calculation also the sibling II-2 who was reported to have symptoms since he was about 20 years old. Age at first symptoms was 40,5 ± 13,3 years in male and 47 ± 6 years in female. The mean time lag between early symptoms and diagnosis was 7,4 ± 3,7 years in living siblings, while the reported disease duration was 34 years in the sibling II-2.

**Table 1:** **Main demographic and clinical data of GSD II sibling*s***

	**II**-**1**	**II**-**4**	**II**-**5**	**II**-**6**	**II**-**7**	**II**-**8**	**II**-**9**	**II**-**10**	**II**-**12**	**II**-**13**
Sex	M	F	F	M	M	F	M	M	F	M
Age 1	56	50	53	54	40	47	38	-	39	35
Age 2	66	59	57	57	53	51	50	-	44	42
MMT-MRC (%)	94	79	82	97	76	86	74	100	78	75
GSGCA(X/27)	25	12	12	25	7	9	9	26	8	6
6MWT (m)	580	297	225	600	324	325	254	610	268	254
CK	170	404	255	140	582	565	404	110	353	887
FVC %	12,8	35	46	11	17	18	20	8	21	18
DBS	3.95	2.70	0.12	4.1	0.11	0.04	0.09	20	0.12	0.31
Muscle GAA		4.2	2.5		2.6	2.3	2.4		2.5	3.1
Cereb.Anom.	+	+	+	+	+	ND	+	-	+	+

The ID number of the patients was relative to the pedigree showed in Figure 2. Age 1: Age at first symptoms (years); Age 2: Age at diagnosis (years); *MMT*-*MRC* (%): manual muscle testing (*MMT*) using the *MRC* (Medical Research Council) grading scale; *GSGCA*: Gait, Stairs, Gowers, Chair, Arms; *MWT*: six-min walk test; *CK*: creatine kinase (50–190 U/L); *FVC* %: forced vital capacity (drop from sitting to supine); *DBS*: *GAA* activity (1,86-21,9 mol/h/l; Muscle *GAA*: *GAA* activity in muscle tissues (34 – 138 pmol/min/mg protein); *CA*: Angio *MR* cerebral anomalies.

With + is indicated the presence of anomalies; with - is indicated the absence of anomalies; ND: not determinate.

The neuromuscular evaluation showed paraspinal muscles weakness with lumbar hyperlordosis in all affected siblings combined with proximal limb muscle weakness (moderate to severe in pelvic and slight to moderate in scapular girdles), waddling gait, winging scapula and positive Gower’s sign in 7 (II-4, 5, 7, 8, 9, 12, and 13). Tongue and velo-pharyngeal muscles involvement with nasal speech were present in 2 siblings (II-1 and II-6) who referred occasional swallowing difficulties. The severity of muscle weakness varied between the siblings regardless of gender and duration of disease. A significant relationships was calculated, in all affected siblings (II-1, 4, 5, 6, 7, 8, 9, 12, 13), comparing age at onset with muscle function impairment scored by GSGC (p < 0.005; R^2^0,8) and with skeletal muscle strength scored by MMT using the MRC grading scale (p < 0.006; R^2^0,7). Neuromuscular exam was normal in sibling II-10.

Serum CK, LDH, ALT, and AST were increased by 1.5-4 times the normal values in all patients, apart from two II-1 and II-6; all other blood parameters were normal, apart from anti-thyroglobulin and anti-peroxidase antibodies titers which were elevated (229.2 UI/mL and 3000 UI/mL) in II-8. Cardiac alterations were not found. FVC in the upright position was ≥80% of the predicted normal in 4 out of 10 siblings (II-1, 6, 8, 10), thus indicating pulmonary function within normal limits, whereas the other 6 (II-4, 5, 7, 9, 12, 13) had FVC upright values ranging 51-79% which dropped in the supine position as compared to normal values, thus indicating a restrictive respiratory disease ranging from a mild to a moderately severe degree, according to the American Thoracic Society standards [19]. Differences in FVC from upright to the supine positions varied less than 10%, in siblings II-6 and II-10, as observed in individuals without restrictive respiratory impairment, whereas they ranged 11–26% in the remaining 8 siblings (II-1, 4, 5, 7, 8, 9, 12, 13), thus indicating the presence of varying degrees of diaphragmatic weakness. A significant relationships (p < 0.05; R^2^0,7) was calculated comparing the age at onset and respiratory function impairment scored by FVC in siblings II-4, 5, 7, 8, 9, 12, 13.

EMG revealed a myopathic pattern characterized by reduced amplitude and duration of motor unit potentials in all muscles explored and pseudomyotonic discharges registered in paraspinal muscles in 9 siblings (II-1, 4, 5, 6, 7, 8, 9, 12, 13) and in tibialis anterior muscle of 3 of them who complained of a steppage and waddling gait.

Muscle biopsy showed the typical pattern of a vacuolar myopathy with intralysosomal glycogen accumulation of variable severity in 6 (II-4, 5, 7, 9, 12, 13) out of 7 patients who underwent muscle biopsy. Vacuolated muscle fibers counted on 3 different histological sections of each muscle specimen ranged from 10 to 100% of the total, without correlation with duration of the disease, age, and gender. Muscle biopsy of II-8 disclosed minor changes with minimal accumulation of glycogen and lysosomes surrounding the paracentral nucleus in only few fibers.

Residual α-glucosidase activity was markedly reduced on both dried blood spots (0.04 – 4.1 mol/h/l; normal range 1.86-21.9 mol/h/l) and muscle tissues at values less than 12% of controls (2.3 - 4,2 pmol/min/mg protein; normal range 34 – 138 pmol/min/mg protein).

*WB*-*M*-*MR of skeletal muscle* was performed in 8 affected siblings (II-1, 4, 5, 6, 7, 9, 12, 13) while the sibling II-8 could not be examined carrying a paramagnetic prosthesis. A mild fatty infiltration and atrophy of adductor magnus, semimembranous, and semitendinosus, long head of the biceps femoris, vastus intermedius, and medialis as well as of the lumbar and at a lower degree of the dorsal paraspinal muscles were observed in 6 siblings (II-4, 5, 7, 8, 12, 13). Muscle involvement was usually symmetrical with the exception of II-5 who showed unilateral atrophy of adductor longus of the thigh. The remaining two patients (II-1, 6) disclosed only a mild fatty infiltration and thinning of paraspinal muscles as well as tongue atrophy.

At MR Angiography dilation of intracranial internal carotid was observed in 8 siblings examined (II-1, 4, 5, 6, 7, 9, 12, 13), whereas basilar artery dolichoectasia was found in 6 (II-4, 5, 7, 9, 12, 13) of them.

DXA examination disclosed osteoporosis in the sibling II-4, osteopenia in II-5, 8, and 13 and it was normal in the other affected and not affected siblings.

### *GAA* mutation analysis

**Figure 3:**
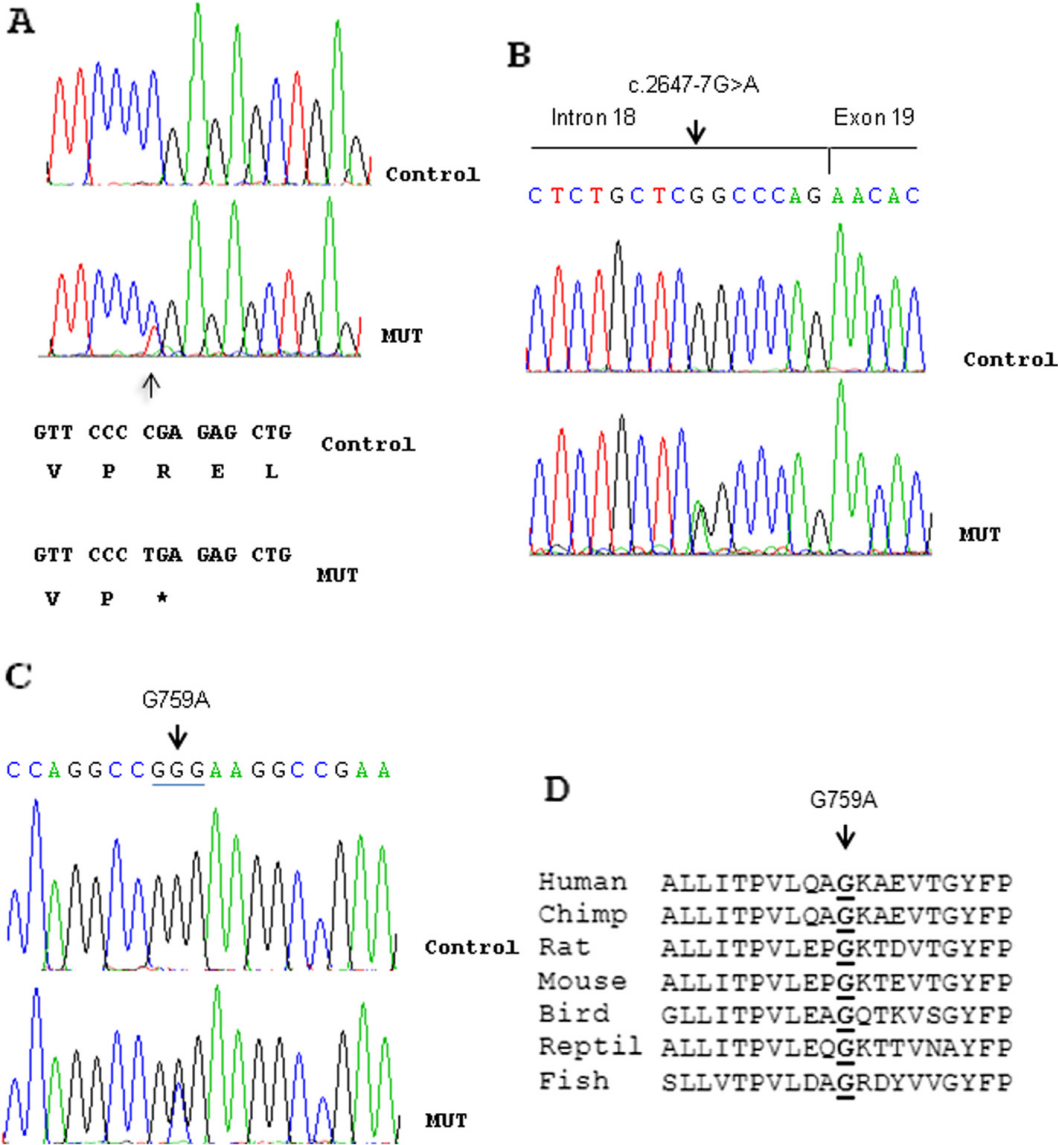
**Mutation analysis of GAA gene. A** to **C** Results of sequencing analysis and detection of mutations in exon 2 (c.118C > T [p.R40X]), intron 18 (c.2647-7G > **A** [p.N882fs]) and exon 16 (c.2276 G > **C** [p.G759A]). Arrowheads mark the site of base alternations. The sequences of an unaffected sample and an affected subject from the Pompe-affected family are reported. The coding triplets and corresponding amino acids decided according to GAA cDNA are indicated. **D** Evolutionary conservation of the glycine residue at position 759 was indicated with an arrow.

In the DNA sample of the index case (II-7) a nonsense mutation (c.118C > T) was identified in exon 2. This mutation, already described in literature (http://www.pompecenter.nl), substitutes an arginine with a stop codon at amino acid 40 (c.118C > T [p.R40X]), and segregates in heterozygous in 7 out of 10 examined siblings (Figure 3A). No further coding mutations were found in this patient, whereas sequence analysis revealed a heterozygous variant in the last 7 nucleotides of intron 18 (c.2647-7G > A) (Figure 3B).

By means of a splicing prediction program (http://www.umd.be/HSF/) a 43% probability was calculated that the c.2647-7G > A change may create a new canonical splicing site in intron 18. Failure in identifying this change in anyone of 200 individuals from healthy population as well as its absence in the dbSNPs (http://www.ncbi.nlm.nih.gov/projects/SNP/) and 1000Genome (http://www.1000genomes.org) databases, suggested this change (c.2647-7G > A) to be a novel mutation of *GAA*. This alternative splice-site inducing change segregated in heterozygous in all 9 examined affected siblings of the family (Table 2). Siblings II-1 and II-6, only carrying the c.2647-7G > A mutation, were investigated for further changes of the *GAA* gene. By sequencing all exons of *GAA*, *a* novel change c.2276 G > C in exon 16, was found in heterozygous in both of them, (Figure 3C). This change causes a glycine to alanine amino acid substitution at position 759 (c.2276G > C [p.G759A]), it is absent in 200 samples from healthy population and it is not reported in the dbSNPs and 1000 Genome databases. Segregation analysis in the sibship showed that apart from II-1 and II-6 this novel mutation was present in simple heterozygous in one further sibling (II-10) only, who is healthy. Evolutionary conservation analysis showed that the glycine at position 759 is highly conserved in all species (Figure 3D), thus supporting a functional role for this mutation.

**Table 2:** **Genotype** –**phenotype correlation of GSD II siblings**

	**II-1**	**II-4**	**II-5**	**II-6**	**II-7**	**II-8**	**II-9**	**II-12**	**II-13**
c.118C > T[p.R40X]		+	+		+	+	+	+	+
c.2647-7G > A [p.N882fs]	+	+	+	+	+	+	+	+	+
c.2276G > C [p.G759A]	+			+					
ICA dilation	+	+	+	+	+	+	+	+	+
Paraspinal muscle involvement	+	+	+	+	+	+	+	+	+
LG-like + respiratory myopathy		+	+		+	+	+	+	+
Dolichoectasia of Basilar artery		+	+		+	+	+	+	+
Tongue weakness	+			+					
Nasal speech	+			+					

The ID number of the patients was relative to the pedigree showed in Figure 2: *ICA* = Internal Carotid Artery; *LG* = limb-girdle.

With + is indicated the presence of mutations and/or symptoms.

### Expression analysis of the c.2647-7G > A mutation

**Figure 4:**
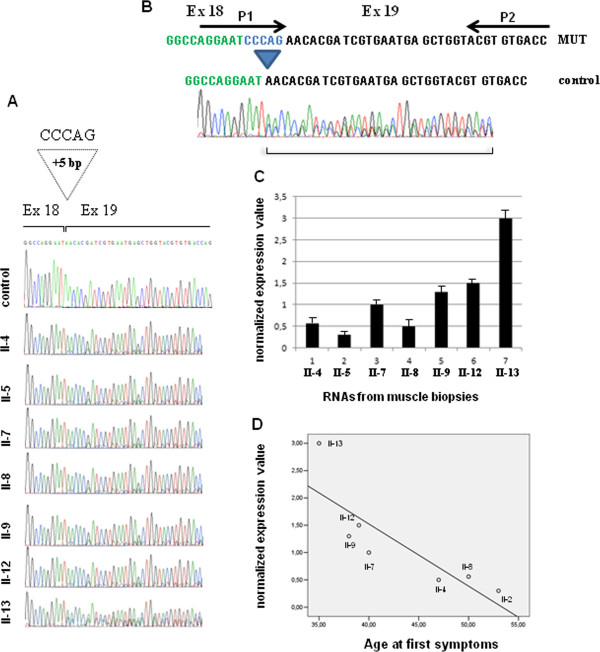
**Expression analysis of the aberrant transcript obtained by the c**.**2647-7G** **>** **A mutation. A** Results of sequencing analysis and detection of intron 18 mutation (c.2647-7G > A [p.N882fs]) in RNAs from muscle biopsies. The mutation inserted 5 bp of the intron 18 in the cDNA sequence producing a frame shift. **B** Schematic representation of the primers (P1 and P2) used in real time experiment to detect only the aberrant transcript. **C** The histogram shows the normalized expression value of the aberrant transcript in RNA of muscle biopsies of the patients carrying the c.2647-7G > A [p.N882fs] mutation. **D** Linear regression analysis using SPSS 13.00 software between the age of onset of the disease and the normalized expression value of the aberrant transcript in muscle biopsies. The circles were numbered corresponding to the pedigree number.

Canonical and alternative splicing mRNAs were found in 7 skeletal muscle biopsies from our patients, confirming that the c.2647-7G > A mutation causes a splicing defect while only the canonical transcript was present in control mRNA (Figure 4A). The mutated was less than the normally spliced transcript in all patients ranging from 20% to 40% of the total but always at lower level than the normal allele (Figure 4A). This data confirmed that the c.2647-7G > A mutation creates a novel acceptor splice site in intron 18. The mutated transcript has 5 nucleotides (CCCAG) of the intron 18 in the RNA sequence producing a frame shift which causes a premature truncation of the protein (c.2647-7G > A [p.N882fs]) (Figure  4A and B). Quantitative mRNA expression assay (qRT-PCR), specific for the mutated allele, confirmed an inter-individual variable expression of the mutant transcript (Figure 4B and C).

Linear regression analysis, performed using SPSS 13.00 software, showed a significant correlation (R^2^ = 0.7; p < 0.01; R^2^ = 0.6; p < 0.05) between the normalized expression value level of the mutated transcript in muscle biopsies, of those patients carrying p.R40X/p.N882fs mutations, with the age of onset of the disease and with GSGC score respectively (Figure 4D).

## Discussion

The present study was aimed to define as accurately as possible the phenotype and the complete coding sequence of *GAA* in affected and healthy individuals of a very large Italian pedigree with adult onset GSDII.

Several genotype/phenotype correlation studies in GSDII patients have well established that a) GAA residual activity of less than 3% the normal is highly recurrent in the classic infantile form whereas it neither falls below this threshold nor exceeds 35% of control values in late-onset patients; b) certain combinations of *GAA* mutations invariably cause the classic infantile form and others the less severe ones [1]; c) *GAA* mutations modify the levels of GAA residual activity in a constant and reproducible way when assessed *in vitro*, but their expression is less well predictable in vivo [1]. In late-onset GSDII patients, indeed, the levels of GAA enzyme activity and the severity of clinical pictures may be highly variable between individuals, even in those who harbor the same combination of *GAA* mutations [7,29]. To explicate the lack of a direct correlation between *GAA* mutations and clinical phenotypes a role has been hypothesized of different bio-molecular events which cooperate in translation, folding, transport ,and maturation of GAA, as well as in regulating the carbohydrates energy metabolism of the body. This “genetic background” might modulate the effect of the combinations of *GAA* mutations on the disease phenotype together with environmental agents and individual habits. However, it has not been possible to verify this hypothesis until now, due to the lack of large informative families, at least in series of Caucasian patients.

In the family herein described, both the clinical-pathological phenotype and the biochemical and molecular features supported the diagnosis of late onset GSDII. The molecular analysis showed that 3 different mutations segregate in the siblings of the second generation. In 7 out of 10 examined siblings, the disease phenotype is linked to the mutation p.R40X which generates a null allele combined with the frameshift mutation p.N882fs which has not been reported previously. p.R40X has already been described in homozygous in two infantile patients and in heterozygous in two unrelated late onset French patients, combined with the common IVS1-13T > G mutation [29,30]. Two further siblings (II-1, II-6) disclosed the same novel mutation p.N882fs but combined with a further novel missense mutation p.G759A on the other GAA allele. The remaining affected sibling (II-2) had died at the time of our observation and neither clinical examination nor genotyping were possible. This patient differs from all other siblings mainly for earlier disease onset. Indeed, his siblings reported he was taken to medical observation for muscle problems first at the age of 20. We have neither explanation for the early disease onset nor we know the exact cause of his death. Indeed, he was followed up by an expert team of physicians for several years and he was considered not to need mechanically assisted ventilation. Therefore a cause for his sudden death different from Pompe disease cannot be excluded with certainty.

The p.G759A mutation was also found in simple heterozygous in one healthy sibling (II-10).

Taking into account the different disease phenotypes which we have observed in our sibship a marked inter-individual clinical heterogeneity seems to emerge. However, the genotype-phenotype correlation allows to associate specific disease features to different mutation compound in siblings. In fact, internal carotid artery dilation and paraspinal muscles involvement were present in all affected siblings who all share the p.N882fs mutation. These features were selectively associated with either basilar artery dolichoectasia and involvement of limb-girdle and respiratory muscles, or with tongue weakness and nasal speech, in those siblings who harbored the combination of p.R40X/p.N882fs and p.N882fs/p.G759A mutations, respectively (Table 2).

Cerebrovascular anomalies have been quite frequently described in recent studies on both sporadic and familiar cases of late-onset GSDII, however they do not seem to occur in all patients [31]. Our data and those of the literature support the hypothesis that cerebral-vascular anomalies in late-onset GSDII patients may be related to specific combination of GAA mutations, however further studies on large series are advisable.

The pattern of distribution of the disease phenotypes in our family suggests that in GSDII patients with a common genetic background the effects of the combination of *GAA* mutations may prevail on demographic factors and individual habits. These latter, however, together with the selective vulnerability of the affected tissues to the derangement of GAA dependent glycogen metabolism, likely modulate the severity of the disease phenotype. Moreover, the importance of non genetic factors in modifying the disease phenotype is suggested by the findings in the sibling II-8, who assumes levotyroxine from several years due to autoimmune-thyroiditis induced hypotyroidsm. Results of clinical, muscle biopsy and *GAA* mutation expression studies disclosed definite differences with respect to the measurements performed in her siblings who harbor the same combination of *GAA* mutations.

The importance of thyroid hormones as regulators of skeletal muscle metabolism and gene expression is well known and their likely role as genotype/phenotype modifying factors in GSDII deserves to be evaluated [32,33].

The novel missense mutation p.G759A is a mild variant which combined with the N882fs splicing mutation contributes to determine a peculiar mild disease phenotype, in two siblings. Most interestingly, we have found this p.G759A mutation together with a null allele mutation in an unrelated late-onset GSDII patient who shows involvement of oral and pharyngeal muscles associated with a moderate degree of weakness of limb girdle and respiratory muscles (data not published).

Age at onset of the disease, distribution, and degree of muscles involvement and rate of progression of the clinical picture varied strikingly between the affected members of our family who carried the p.R40X/p.N882fs mutation. Studying muscle changes on H&E stained sections did not allow us to find any relationship between degree of muscle vacuolation and age, gender and duration of the disease. Conversely, preliminary quantitative expression studies on these siblings show that this phenotype heterogeneity may be partially explained by inter-individual variable expression of the mutant p.N882fs transcript.

The most relevant finding in our study is the occurrence of three mutations, two of which being previously unreported, distributed in a very large sib-ship, which represents an informative cohort to follow the phenotype effects of different mutational complexes in presence of reduced inter-individual differences in the genetic-background. The pattern of transmission of p.R40X and p.G759A mutations strongly suggest they were both carried by one parent, likely the father, reported to have died for respiratory failure at 69 years of age. The occurrence of 3 different mutations in the first generation of our pedigree could explain the very high incidence of affected individuals in the same sib-ship, which gives the wrong suggestion of a dominant transmission. Similarly 3 different mutations of *GAA* in non-consanguineous parents have already been reported in a pedigree in which the father carrying 2 mild mutations (p.Asp404Asn and c.-32-13T > G) did not have any outward manifestation of late onset Pompe disease and the healthy mother carried the p.Leu811fs mutation alone [34]. They were genetically investigated only after the birth of their first child who showed the classic infantile Pompe disease. Furthermore, patients have been published, showing a mild late-onset disease phenotype, in whom only one mutation has been identified [35,36]. These cases may be explained by the presence of a null allele and/or a regulative mutation or by lacking sequencing of all *GAA* exons.

## Conclusion

This work report the largest informative family with late-onset Pompe disease described in the literature showing a peculiar complex set of mutations of *GAA* gene that partially elucidate the clinical heterogeneity of this family.

Nowadays, a complete molecular analysis of the *GAA* gene of affected individuals is highly recommended either for a better understanding of the genotype-phenotype relationships or, more importantly, to have the opportunity to be prepared to administer future selective therapies based on the specificity of GAA mutations.

## Competing interests

All authors declare that they have no competing interests.

## Authors’ contributions

SS, OF, DD, FC, and GDI recruited the family described herein, collected the clinical data and performed neurology, electrophysiology and myopathology studies. GC, MC and LDV performed the clinical MR imaging studies. TE, DF and FG performed the sequencing and expression analyses. AT and CA performed GAA enzyme measurements and genomic DNA screening for known *GAA* mutations. SS, TE and GDI oversaw all aspects of the research. SS, TE and GDI initiated, planned and coordinated the study. SS and TE wrote the manuscript. AT and CA have critically revised the manuscript. All authors read, edited and approved the final version of the manuscript.
